# Optimization of Two Methods for the Rapid and Effective Extraction of Quinine from *Cinchona officinalis*

**DOI:** 10.3390/plants14030364

**Published:** 2025-01-25

**Authors:** Gianella Ochoa, Leonardo Armijos, Jorge G. Figueroa, Ximena Jaramillo-Fierro, Natalí Solano-Cueva

**Affiliations:** 1 Carrera de Ingeniería Química, Facultad de Ciencias Exactas y Naturales, Universidad Técnica Particular de Loja, París s/n y Praga, Loja 110107, Ecuador; geochoa1@utpl.edu.ec (G.O.); rlarmijos2@utpl.edu.ec (L.A.); 2 Departamento de Química, Facultad de Ciencias Exactas y Naturales, Universidad Técnica Particular de Loja, París s/n y Praga, Loja 110107, Ecuador; jgfigueroa@utpl.edu.ec (J.G.F.); nesolano@utpl.edu.ec (N.S.-C.)

**Keywords:** *Cinchona officinalis*, quinine, microwave-assisted extraction, ultrasound-assisted extraction, HPLC-DAD-ESI-IT-MS

## Abstract

This study successfully optimized two advanced extraction methods, microwave-assisted extraction (MAE) and ultrasound-assisted extraction (UAE), for the efficient and rapid recovery of quinine from *Cinchona officinalis*. Among the evaluated parts of the plant, the bark consistently yielded the highest quinine concentration, highlighting its significance as the primary source for alkaloid extraction. The optimized conditions for MAE (65% ethanol, 130 °C, 34 min) achieved a maximum yield of 3.93 ± 0.11 mg/g, while UAE (61% ethanol, 25 °C, 15 min) provided a faster but slightly lower yield of 2.81 ± 0.04 mg/g. These findings confirm the superiority of MAE and UAE over conventional methods like Soxhlet extraction in terms of time efficiency and sustainability. The quantification of quinine using high-performance liquid chromatography (HPLC) coupled with advanced detection methods further validated the reliability and reproducibility of the results. While this study focused on optimizing extraction and quantification parameters, it sets the groundwork for future research into the sustainable utilization and potential valorization of *C. officinalis* byproducts. These findings not only provide a standardized protocol for extracting quinine but also contribute to the broader application of green chemistry principles in pharmaceutical production.

## 1. Introduction

*Cinchona officinalis*, commonly known as cascarilla, is a plant of great historical and medicinal importance, famous for its content of quinine, an alkaloid with antimalarial and analgesic properties [1,2,3]. Native to the Andean forests stretching from Venezuela to Bolivia, quinine has been used for centuries as one of the most effective treatments against malaria [4]. The use of *Cinchona* bark by indigenous people of South America to treat the symptoms of malaria dates to the 17th century, when the plant was known as the “fever tree” [5]. In the early 19th century, quinine was first isolated by French chemists Pierre-Joseph Pelletier and Joseph-Bienaimé Caventou, marking a milestone in pharmacology and consolidating it as the standard treatment for malaria during the 18th and 19th centuries, especially in Europe [6]. Quinine’s relevance lies not only in its therapeutic efficacy but also in its historical role as one of the first examples of how natural compounds can be transformed into essential medicines [7,8,9]. Although largely superseded by synthetic drugs in the 20th century, interest in *C. officinalis* and quinine has resurged in recent years, especially due to its potential in treating respiratory diseases and quinine’s continued usefulness in regions where resistance to newer drugs is a growing problem [10,11,12]. This underscores the importance of continuing detailed studies on optimizing its extraction and quantification to ensure the quality and efficacy of plant-derived pharmaceuticals, reaffirming quinine as a highly relevant drug in the fight against infectious diseases.

The extraction of active ingredients from plants is an important step in obtaining bioactive compounds for pharmaceutical applications, and the efficiency of this process can significantly influence the quality and quantity of the recovered compounds. Traditionally, methods such as maceration, percolation, and infusion have been used for the extraction of alkaloids and other secondary metabolites [13]. However, these methods may be limited by low selectivity, degradation of heat-sensitive compounds, or the need for large solvent volumes, complicating subsequent concentration and purification processes [14]. To overcome these limitations, modern extraction techniques have been developed that aim to improve the efficiency, selectivity, and sustainability of the process.

Among the various extraction techniques, microwave-assisted extraction (MAE) and ultrasound-assisted extraction (UAE) have emerged as efficient and sustainable alternatives to traditional methods such as Soxhlet extraction. MAE leverages microwave energy to disrupt plant cell walls, significantly enhancing the release of bioactive compounds like quinine while reducing extraction time to minutes compared to the ~8 h required by Soxhlet [15]. UAE, on the other hand, uses ultrasonic waves to create cavitation, improving solvent penetration and facilitating efficient mass transfer within the plant matrix [16]. In contrast, Soxhlet extraction is limited by its prolonged duration, high solvent consumption, and potential thermal degradation of sensitive compounds, making it less favorable for high-throughput applications [17,18]. These advantages highlight the suitability of MAE and UAE for the rapid and efficient extraction of quinine, aligning with the study’s focus on optimizing modern extraction techniques for *C. officinalis*. Additionally, in this study, response surface methodology (RSM) was used to optimize the extraction conditions, allowing the identification of the optimal solvent, temperature, and extraction time, thus maximizing the recovery of the compound and ensuring the applicability of the analytical method under various conditions [19,20].

Regarding the characterization of active ingredients, it is essential for determining their purity, concentration, and composition. Traditional methods, such as UV-visible spectroscopy [21] and thin-layer chromatography (TLC), have been used for the preliminary analysis of alkaloids in plant extracts [22]. Advanced techniques like gas chromatography-mass spectrometry (GC-MS) offer high sensitivity for volatile compounds but require derivatization for alkaloid analysis [14]. High-performance liquid chromatography (HPLC), however, has emerged as the preferred technique for quantifying non-volatile bioactive compounds, including quinine, without derivatization [23,24,25,26]. The use of HPLC and GC-MS has allowed the identification and characterization of secondary metabolites in *C. officinalis*, including alkaloids, phenols, flavonoids, and terpenoids, highlighting the remarkable therapeutic potential of the extracts of this species [27].

Several methods for the extraction and quantification of quinine from *Cinchona* species have been reported in the literature, showcasing the importance of adjusting extraction protocols to the unique properties of each species [28]. Misra et al. (2008) [29] optimized quinine extraction conditions using high-performance thin-layer chromatography (HPTLC) coupled with UV detection, achieving reliable quantification of alkaloids from *Cinchona* bark extracts. Extraction was performed using the Soxhlet technique with 10 g of powdered bark (14 mesh) and 200 mL of solvent in a water bath for 10 h. Methanol modified with 20% (*v*/*v*) diethyl amine was identified as the optimal solvent, yielding the highest recovery of quinine (2.202% by dry weight). The extracts were concentrated in vacuo and redissolved in methanol for analysis. Similarly, Hariyanti et al. (2021) [30] employed the Soxhlet method to extract quinoline alkaloids from *Cinchona succirubra* in cosmetic formulations, highlighting the importance of physical stability and safety of the resulting products. In addition, other studies have explored different approaches for the extraction of quinine and related bioactive compounds. Granados-Chinchilla and Campos-Arguedas (2024) [31] described methods of extracting quinine and chlorophyll from *Cinchona pubescens* bark using solid–liquid and liquid–liquid extraction techniques, highlighting the utility of these techniques in educational settings and as tools for fluorescence analysis. Fan et al. (2021) [32] developed hydrophobic natural alcohol-based deep eutectic solvents (NADES), which demonstrated high efficiency for quinine extraction compared to traditional organic solvents such as trichloromethane and ethyl acetate, offering a sustainable and biocompatible alternative.

Additionally, recent advancements in spectrophotometric and electroanalytical methods have been employed for quinoline-based compounds, highlighting their efficiency in detecting and quantifying compounds like quinine at low concentration levels, with minimal interference [33]. These works underline the importance of tailoring extraction protocols to the specific characteristics of the *Cinchona* species and using precise analytical methods for quantification. This study builds on these findings by focusing on MAE and UAE, which have demonstrated faster and more efficient extraction compared to traditional Soxhlet extraction [18]. Through the optimization of UAE and MAE extraction parameters—including solvent composition, temperature, and time—and refining the HPLC quantification process, this research aims to establish a comprehensive protocol for the efficient extraction and precise quantification of quinine. Such an approach not only ensures reproducibility and accuracy in the extraction process but also aligns with the principles of sustainability, supporting the responsible utilization of *C. officinalis* for pharmaceutical applications.

## 2. Results

### 2.1. Quantification of Quinine

In this study, the analysis of the initial concentrations of quinine in different parts of the *C. officinalis* species collected from the three locations revealed that the alkaloid was most concentrated in the bark. In contrast, no quinine was detected in the leaves, as shown in Table 1.

Complete quantification considering the location and plant part was performed only with MAE as a preliminary assay. This approach was justified because MAE has demonstrated greater efficiency and reproducibility in quinine extraction in different plant matrices, as observed in this study. Based on these findings, the material was selected to continue with the optimization of the extraction process with both MAE and UAE. Although the bark sample from Tapichalaca yielded the highest quinine concentration (6.30 ± 0.19 mg/g), the optimization of the MAE and UAE methods was conducted using samples from Villonaco. This decision was based on the greater availability of plant material from this location, which ensured sufficient quantities for comprehensive optimization studies. The bark from Villonaco, with a quinine concentration of 3.93 ± 0.11 mg/g, provided a suitable alternative for optimizing the extraction methods while maintaining methodological rigor.

### 2.2. Optimization of the Extraction Process

In the optimization process, the maximum values of temperature, time, and EtOH concentration used were 150 °C, 60 min, and 100%, respectively. As shown in Table 2, the quinine content varied between 0.14 mg/g and 3.62 mg/g under the different conditions evaluated.

**Figure 1:**
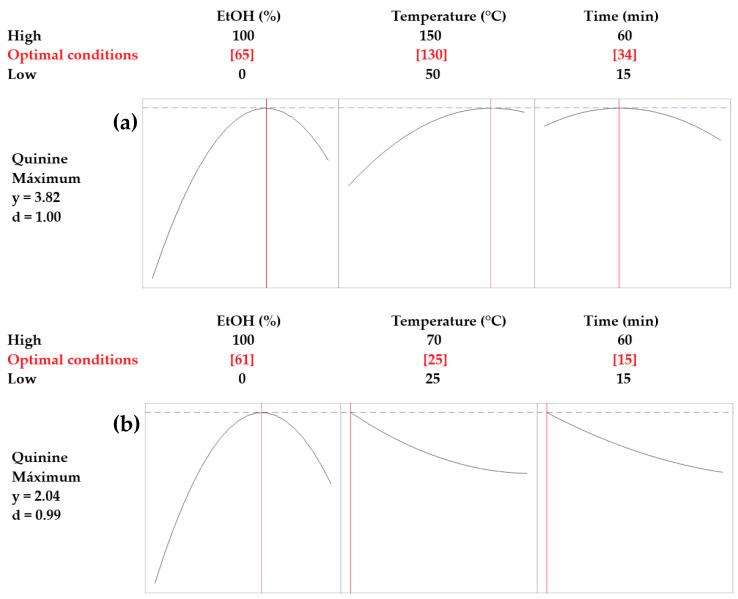
Optimal conditions for extraction of quinine from *C. officinalis* by (**a**) microwave-assisted extraction and (**b**) ultrasound-assisted extraction. y represents the maximum quinine concentration obtained under the optimized conditions; d corresponds to the desirability value, where 1 indicates the most favorable outcome, and lower values reflect less optimal results. Red numbers indicate the optimized conditions for quinine extraction, while black numbers represent the ranges of conditions evaluated during the study.

Figure 1 shows the results of the optimization of the quinine extraction process from *C. officinalis*. The optimal conditions determined for the microwave-assisted extraction were a 65% aqueous EtOH solution, a temperature of 130 °C, and an extraction time of 34 min. Under these conditions, the highest concentration of quinine was obtained in the bark, reaching 3.82 mg/g quinine in dry weight. On the other hand, the optimal conditions determined for the ultrasound-assisted extraction were a 61% aqueous EtOH solution, a temperature of 25 °C, and an extraction time of 15 min. Under these conditions, the highest concentration of quinine was obtained in the bark, reaching 2.04 mg/g quinine in dry weight.

**Figure 2:**
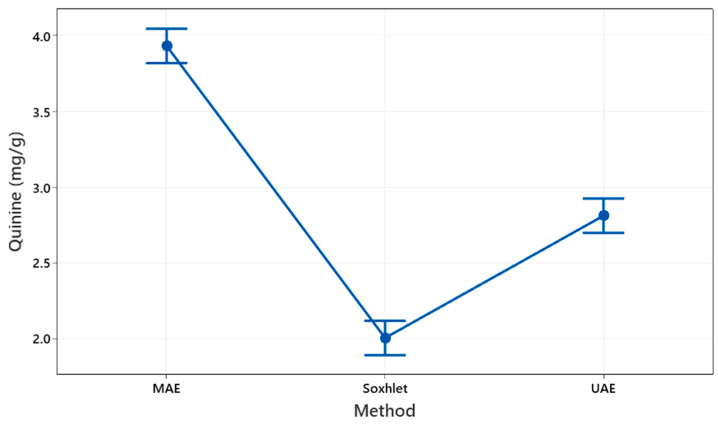
Quinine yield by extraction method with 95% confidence intervals.

In addition, an analysis of variance (ANOVA) was conducted to evaluate the impact of independent variables—EtOH concentration, temperature, and time—on quinine extraction. Tukey’s post hoc test revealed significant differences in quinine yields between MAE, UAE, and Soxhlet extraction, with MAE showing the highest yield (3.93 ± 0.11 mg/g), followed by UAE (2.81 ± 0.04 mg/g) and Soxhlet (2.01 ± 0.07 mg/g). These results underscore the superior efficiency of MAE under optimized conditions. Figure 2 shows quinine yields for each extraction method (MAE, UAE, and Soxhlet), with 95% confidence intervals calculated using the pooled standard deviation.

For both MAE and UAE, the results showed that several variables had a statistically significant effect (*p* < 0.05), as detailed in Table 3. The Pareto Chart of standardized effects confirmed that factors exceeding the threshold of 2.57 were significant. Specifically, in MAE, most independent variables were impactful, while in UAE, ethanol (EtOH) concentration exhibited significant linear and quadratic effects, and the interaction between time and temperature also had a notable influence. The coefficients of determination indicated that the models explained 96% and 98% of the variability in MAE and UAE, respectively.

The regression equations in uncoded units that allowed the prediction of quinine concentration under different extraction conditions are as follows:

MAE:

(1) $$\begin{array}{c} Quinine\left(mg/g\right)=-0.63+0.0714E+0.0296T+0.0122t-0.000763E\times E-0.000219T\times T-0.000900t\times t+\\ 0.000218E\times T-0.000051E\times T+0.000403T\times t \end{array}$$

UAE:

(2) $$\begin{array}{c} Quinine\left(mg/g\right)=1.671+0.04929E-0.0393T-0.0292t-0.000395E\times E+0.000243T\times T+0.000140t\times t-\\ 0.000023E\times T-0.000047E\times t+0.000406T\times t \end{array}$$

where E, T, and t represent EtOH concentration, temperature, and time, respectively.

**Figure 3:**
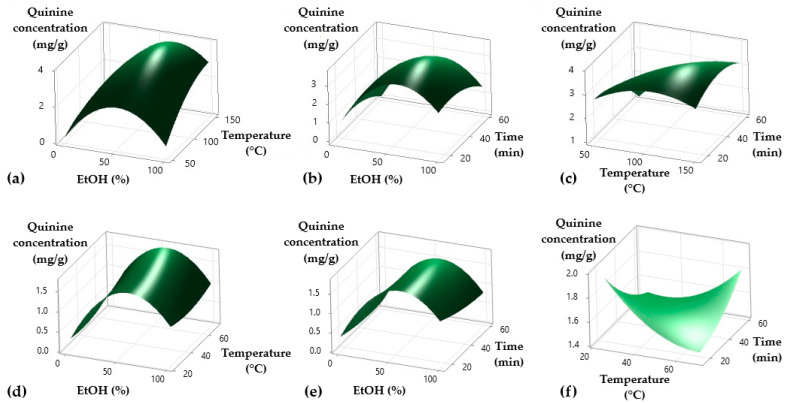
Three-dimensional response surface graphs for quinine extraction optimization by (**a**) microwave-assisted extraction (**a**–**c**) and ultrasound-assisted extraction (**d**–**f**).

The study of the three-dimensional response surface graphs illustrates how quinine concentration varies when two independent variables are evaluated simultaneously in the extraction processes. For the MAE process, the interaction between EtOH concentration and temperature showed that quinine concentration increased significantly with 55% EtOH and a temperature of 140 °C (Figure 3a). Similarly, when EtOH concentration and extraction time interacted, the maximum quinine concentration was achieved with 55% EtOH and a time of 50 min, though the effect of time was less pronounced compared to temperature (Figure 3b). Finally, the interaction between temperature and time revealed that the highest quinine concentration was obtained at 120 °C and 50 min (Figure 3c). These results highlight the fundamental role of optimizing temperature and EtOH concentration to maximize quinine extraction efficiency in MAE. For the UAE process, the interaction between EtOH concentration and temperature indicated an optimal quinine concentration at 50% EtOH and 45 °C (Figure 3d). Similarly, the interaction between EtOH concentration and time showed the highest concentration with 50% EtOH and 30 min (Figure 3e). Finally, the interaction between temperature and time revealed a maximum quinine concentration at 40 °C and 25 min (Figure 3f). These findings underscore the importance of moderate conditions in UAE to ensure efficient quinine extraction while avoiding degradation or loss of yield due to extreme conditions. This comparative analysis demonstrates that both extraction methods, MAE and UAE, require carefully balanced parameter settings to achieve optimal quinine extraction, with MAE favoring higher temperature conditions and UAE excelling under moderate settings.

## 3. Discussion

### 3.1. Quinine Concentration in Different Parts of C. officinalis

In this study, the observed variation in quinine concentration among different parts of *C. officinalis* highlights the biological role of secondary metabolites in plant defense. The bark exhibited the highest concentration of quinine, significantly more than the trunk, while the leaves showed no detectable levels. This distribution aligns with the understanding that bark serves as a key protective layer, rich in defensive secondary metabolites essential for deterring herbivores and pathogens [34]. The quinine concentration obtained from the bark of *C. officinalis* in this study stands out when compared to other studies, which often highlight the bark as the richest source of alkaloids. For example, a recent analysis demonstrated total alkaloid content in the bark of *Cinchona* species ranges from 7 to 12%, with quinine accounting for up to 90% of the total alkaloids [23]. In contrast, the concentration of quinine in the leaves of some *Cinchona* species, such as *C. pubescens*, was reported to be as low as 0.06 µg/g [35]. This stark difference highlights the variability in quinine content between different parts of the plant, with the bark consistently showing much higher yields. Furthermore, while Remuzgo et al. (2020) [35] also identified other alkaloids, such as quinidine and cinchonidine, in proportions comparable to quinine, the overall alkaloid concentrations were notably lower than those found in our study. This supports the notion that the bark of *Cinchona* species continues to be the most effective source for extracting quinine.

Historically, higher concentrations of quinine have been documented. For example, Fonfría et al. (2000) [36] reported that species such as *Cinchona calisaya* and *C. officinalis* had bark with quinine concentrations averaging 38 mg/g, especially in trees cultivated in regions specifically selected for their medicinal properties. This average is notably higher than the values found in this study, 3.93 ± 0.11 mg/g by MAE and 2.81 ± 0.04 mg/g by UAE, which could be attributed to factors such as environmental conditions, plant age, and extraction techniques used in earlier studies. Indeed, several historical studies reporting higher values employed traditional extraction methods that, while less sustainable, were designed to maximize yield. In contrast, the modern techniques used in this study, such as MAE and UAE, prioritize sustainability, reduced extraction times, and the use of more environmentally friendly solvents [28]. While this focus on optimized parameters for efficiency and sustainability may limit yields compared to traditional methods, more recent studies have also reported lower yields, particularly in non-bark parts of the plant. This supports the conclusion that the bark of *C. officinalis* remains the primary and most reliable source of quinine. The variability in results highlights the need for further optimization of extraction protocols to ensure consistency in quinine yields across different studies and conditions.

On the other hand, to analyze the results of Table 1 in the context of the plant collection location, we must consider how the environmental factors specific to each location might influence the concentration of quinine in the different parts of *C. officinalis*. As shown in Table 1, the highest quinine concentration was recorded in the bark collected from Tapichalaca (6.30 ± 0.19 mg/g), followed by the bark from Villonaco (3.93 ± 0.11 mg/g). In contrast, the bark collected from Palanda showed no detectable quinine levels. This significant variability can be attributed to the environmental conditions of each location, including altitude, temperature, soil composition, and sunlight exposure, all of which are known to influence alkaloid biosynthesis in plants. The trunk also exhibited differences, with the sample from Tapichalaca containing a much higher concentration of quinine (1.95 ± 0.05 mg/g) compared to the sample from Villonaco (0.12 ± 0.03 mg/g), while the trunk from Palanda again showed no detectable quinine. These results suggest that the environmental conditions in Tapichalaca are more conducive to quinine accumulation, not just in the bark but also in the trunk, while Palanda appears less favorable for quinine biosynthesis, potentially due to lower exposure to stress factors that trigger alkaloid production. The leaves from all localities, prepared by manual grinding, did not show detectable levels of quinine, confirming its primary concentration in the bark and, to a lesser extent, in the trunk, which were prepared by grinding with an ultracentrifugal mill. These differences across localities emphasize the role of environmental and geographical factors in quinine distribution within *C. officinalis* and highlight the need for site-specific strategies to optimize alkaloid production for pharmaceutical use.

The findings of this study expand the current knowledge by showing that optimized extraction from the bark of *C. officinalis* can yield a relatively high amount of quinine. However, the concentration obtained is still lower than the highest previous records, possibly due to differences in the environmental conditions under which the plants were grown, such as altitude, soil composition, and climate, as well as modern processing techniques, which may prioritize other factors such as sustainability or speed over maximizing yield. This underscores the importance of continued research into optimizing both cultivation and extraction processes for *Cinchona* species to improve quinine yields, especially as demand for plant-derived pharmaceuticals remains high.

### 3.2. Effect of the Solvent and Its Mechanism of Action

In this study, ethanol was chosen as the solvent for extraction because it provides an ideal balance of polarity to extract both hydrophilic and hydrophobic compounds like quinine, which is slightly polar. The ethanol–water mixture enhances penetration into plant cell walls and solubilizes active compounds, maximizing quinine recovery while minimizing the co-extraction of unwanted substances. Additionally, ethanol is a safe, non-toxic solvent recognized as GRAS (generally recognized as safe) by the FDA, making it suitable for pharmaceutical, nutraceutical, and cosmetic applications. Its compatibility with industrial processes further supports its selection over other alcohols like methanol, which are more toxic and less environmentally friendly [37].

During the optimization procedure, EtOH concentrations of 0%, 50%, and 100% were tested to determine the most effective solvent conditions. The optimal extraction of quinine was estimated at a concentration of 61% and 65% EtOH in water, for UAE and MAE, respectively. At these concentrations, EtOH forms a solution of intermediate polarity, ideal for interacting with both types of components, hydrophilic and hydrophobic, in plant tissue [14]. The solvent’s mechanism of action is based on the ability of the homogeneous mixture of EtOH and water to penetrate plant cell walls, solubilize active compounds, and release quinine into the solution. By reducing the surface tension between the solvent and the plant matrix, the EtOH–water mixture improves penetration, enhancing the dissolution of quinine. This reduction occurs because EtOH alters the interactions between water molecules, which reduces cohesion and allows the solvent to more effectively enter the plant matrix. Additionally, the water in the mixture hydrates the plant matrix, which aids in cell opening and the release of active compounds, while facilitating the dissolution of polar compounds within plant cells, ultimately increasing the overall efficiency of the extraction [24]. The EtOH–water mixture also enables selective extraction, where the more hydrophobic compounds, such as quinine, are preferentially extracted due to the ability of EtOH to break hydrophobic interactions within the matrix. This phenomenon increases the recovery of quinine while lowering the co-extraction of other unwanted compounds, which is essential to obtain purer and more concentrated extracts [25].

### 3.3. Effect of Temperature on Extraction

Temperature significantly influences quinine’s solubility, solvent viscosity, and extraction kinetics within the plant matrix. For microwave-assisted extraction (MAE), higher temperatures, such as the optimal 130 °C identified in this study, enhance quinine solubility and diffusion, as predicted by Le Chatelier’s principle. Elevated temperatures also reduce solvent viscosity, improving penetration into cell walls and increasing extraction efficiency [24]. However, exceeding 130 °C can induce decomposition reactions, compromising the extract’s purity [25]. In contrast, ultrasound-assisted extraction (UAE) achieved optimal results at a significantly lower temperature of 25 °C. This lower temperature minimizes the risk of thermal degradation while leveraging ultrasonic cavitation to disrupt cell structures and release quinine effectively [17]. These findings highlight the distinct temperature requirements for MAE and UAE, with each method offering specific advantages depending on the extraction conditions and thermal stability of the target compound.

### 3.4. Effect of Extraction Time on Extraction

Extraction time is fundamental in determining the yield of alkaloids like quinine. Adequate extraction time allows optimal interaction between the solvent and plant matrix, promoting the solubilization and release of active compounds. However, extending the extraction time beyond the saturation point can lead to the co-extraction of unwanted compounds, such as tannins and flavonoids, which can reduce extract purity. Additionally, prolonged extraction at high temperatures can cause oxidative or thermal degradation of quinine [13,38]. In this study, the optimal extraction time varied depending on the method: 34 min for microwave-assisted extraction (MAE) at 1000 W and 15 min for ultrasound-assisted extraction (UAE). The longer extraction time for MAE reflects the method’s reliance on thermal energy to disrupt cell structures, while the shorter time for UAE leverages the efficiency of ultrasonic cavitation to release quinine rapidly. Both times were sufficient to maximize quinine yield without significant degradation or co-extraction of undesirable compounds, showcasing the tailored advantages of each technique in achieving efficient and high-quality extractions [24,39].

### 3.5. Integration of Chromatography and Advanced Techniques in Quantification

Accurate quantification of alkaloids like quinine is essential for ensuring the quality and safety of plant-derived pharmaceuticals. High-performance liquid chromatography (HPLC), particularly when coupled with sensitive detectors such as diode array detection (DAD) and mass spectrometry (MS), is widely recognized for its high resolution, specificity, and ability to quantify compounds in complex matrices. In this study, HPLC-DAD-ESI-IT-MS was used to identify and quantify quinine with optimal precision and accuracy. For example, Remuzgo et al. (2020) [35] demonstrated the effectiveness of HPLC in differentiating alkaloids in *C. officinalis* extracts, underscoring its capacity to provide detailed compositional profiles. Coupling HPLC with MS further enhances detection and quantification, enabling the identification of compounds at low concentrations and providing essential structural information that complements separation data [9,40].

In this study, MAE yielded the highest quinine concentration (3.93 ± 0.11 mg/g), followed by UAE and Soxhlet, with 2.81 ± 0.04 mg/g and 2.01 ± 0.07 mg/g, respectively. These results are comparable and, in some cases, lower or higher than those reported in previous studies, which underlines the variability in quinine extraction depending on the specific conditions of each investigation [41]. To our knowledge, no previous study has directly compared MAE, UAE, and Soxhlet extraction for the recovery of quinine from *C. officinalis*. This study addresses this gap by demonstrating that MAE achieved the highest yield of quinine (3.93 ± 0.11 mg/g) in only 34 min, while UAE provided a yield of 2.81 ± 0.04 mg/g in 15 min. Both methods outperformed Soxhlet extraction, which required 10 h to reach a yield of 2.01 ± 0.07 mg/g. These findings highlight the efficiency of MAE and UAE in terms of time, energy consumption, and compound conservation. Although studies on other bioactive compounds have reported similar trends, such as higher yields and shorter extraction times for MAE and UAE compared to Soxhlet extraction, their application to quinine has not been previously explored. For example, Guglielmetti et al. (2017) [42] reported that EAU was effective in extracting caffeoylquinic acids and caffeine with reduced time and energy usage, while Gatti et al. (2004) [39] demonstrated that MAE preserves bioactive compounds due to its rapid heating mechanism, which minimizes thermal degradation. The results of this study confirm that these advanced techniques can be successfully adapted for the efficient extraction of quinine, providing a more sustainable alternative to traditional methods.

Despite the efficiency and sustainability advantages of the MAE and UAE methods, it is important to recognize that both methods have certain limitations. In the case of MAE, the use of high temperatures and pressures can potentially degrade thermolabile compounds, including alkaloids like quinine, if conditions are not meticulously controlled. Similarly, UAE, while faster and less energy-intensive, may exhibit reduced extraction efficiency for compounds deeply embedded in plant matrices due to limited solvent penetration. Both methods rely on ethanol–water mixtures, which, while environmentally friendly, may not achieve the same extraction yields as traditional organic solvents. Additionally, the scalability of these techniques for industrial applications presents challenges, as factors such as equipment costs and process optimization at larger volumes must be addressed to ensure practical implementation [28].

### 3.6. Potential Impact of Results on Pharmaceutical Production

The results obtained in this study, particularly the identification of an optimal quinine concentration under specific extraction conditions, could have a significant impact on pharmaceutical production. Optimizing extraction conditions, such as EtOH concentration, temperature, and extraction time, allows for improved efficiency of the production process. This not only maximizes the yield of extracted quinine but also contributes to a cost reduction by decreasing solvent and energy consumption. Moreover, optimizing these parameters contributes to the quality of the final product by enhancing the purity and efficacy of quinine extracts, which is fundamental for producing safe and effective medicines. While optimization of extraction conditions plays a significant role, it is one of several factors needed to ensure consistent product quality.

The findings of this study also open the door to the creation of new pharmaceutical formulations that take advantage of the therapeutic properties of quinine. Optimizing extraction facilitates the production of high-purity quinine, which enables its inclusion in more advanced and targeted pharmaceutical formulations, such as controlled-release systems or combination therapies that enhance therapeutic efficacy. This could include the development of new antimalarial drugs that combine quinine with other therapeutic agents to improve treatment efficacy, or the exploration of quinine in the treatment of other diseases, such as certain cardiovascular disorders or inflammatory conditions, where its properties may be beneficial.

Finally, the methodology optimized in this study is fundamental for standardization and quality control in the industrial production of quinine. The ability to consistently reproduce extraction results under the identified optimal conditions ensures that each batch of product maintains the same concentration and purity of quinine, which is important to meeting international regulatory standards. Implementing these quality control practices not only ensures the safety and efficacy of *C. officinalis* derived medicines but also strengthens confidence in natural products as sources of reliable and standardized medicinal therapies.

## 4. Materials and Methods

### 4.1. Reagents

The HPLC standards quinine (purity 97.8%) and caffeine (purity 99.8%) were purchased from Sigma–Aldrich, Buchs, Switzerland and Sigma–Aldrich, St. Louis, MO, USA, respectively. The reagents used in the mobile phase were as follows: for phase A, Optima LC-MS Grade Water was supplied from Fisher Chemical (Pittsburgh, PA, USA), and Formic Acid Optima LC/MS (purity 99.0%) was purchased from Fisher Chemical (Pardubice, Czech Republic); for phase B, HPLC-MS Grade Methanol (MeOH, purity 99.9%) was purchased from Fisher Chemical (Pittsburgh, PA, USA). The solvents used in the extraction process and quantification of quinine were Ethanol Absolute (EtOH, purity 99.9%) received from Kimia (Witham, UK) and ultrapure Water Type I that was purified using a MicroPure ST (Thermo Scientific, Rheinfelden, Germany).

### 4.2. Plant Material and Extract Preparation

The plant material of *C. officinalis* was collected in November 2021 in two provinces in Southern Ecuador: the province of Loja—in the Villonaco sector, Loja canton (WGS84 −4°2′4.0452″ N; −79°15′56.956″ E); and the province of Zamora Chinchipe—in the city of Palanda, Palanda canton (WGS84 −4°39′28.512″ N; −79°8′2.472″ E), and in the Tapichalaca National Reserve in the Valladolid parish, Palanda canton (WGS84 −4°30′20.628″ N; −79°7′34.392″ E). Three parts of the plant were selected for the study: bark, leaves, and trunk. The collected samples were dried in a chamber (DY-110H, Lassele, Seoul, Republic of Korea) at 37 °C (bark and trunk for 4 days, leaves for 1 day) to a humidity of about 12%. Subsequently, the samples were crushed to achieve a uniform average particle size of less than 500 μm using a ZM 200 ultracentrifugal mill (Retsch GmbH, Haan, Germany).

### 4.3. Extraction Process and Its Optimization

The response surface methodology (RSM) was employed to optimize the extraction of quinine from bark using microwave-assisted extraction (MAE) and ultrasound-assisted extraction (UAE). Solvent concentration, extraction time, and temperature were evaluated as independent variables, with the tested conditions detailed in Table 1 (a and b). Additionally, Soxhlet extraction was performed as a reference method following the conditions described by Gatti et al. [39]. To prevent the sample-to-solvent ratio from influencing the results, the same ratio was consistently applied across all three methods.

MAE was conducted using a laboratory microwave oven (Mars 6, CEM Corporation, Matthews, NC, USA) operating at 1000 W, with an initial ramp time of 15 min. Samples of 1 g were weighed into microwave extraction tubes, followed by the addition of 40 mL of EtOH. For UAE, a bath sonicator (FS30D, Fisher Scientific, Waltham, MA, USA) with a frequency of 42 kHz and a power output of 100 W was used. In this method, 25 mg of the sample was weighed into 2 mL microtubes, and 1 mL of the solvent was added. The internal standard, caffeine, was added along with the solvent before starting the extraction process.

The optimal conditions determined were as follows: for MAE, 65% EtOH, a temperature of 130 °C, and an extraction time of 34 min; for UAE, 61% EtOH, a temperature of 25 °C, and an extraction time of 15 min. After extraction, the solid phase was separated from the liquid extract by centrifugation at 8000 rpm for 15 min (Sorvall ST8, Thermo Scientific, Waltham, MA, USA). The liquid extracts were filtered using 0.2 μm hydrophilic PTFE syringe filters (Titan 3, Thermo Scientific, Shanghai, China). The filtered extracts were then transferred to vials for chromatographic analysis.

### 4.4. HPLC-MS Analysis Conditions of Quinine

Development and optimization of the analytical method for the quantification of quinine was carried out by high-performance liquid chromatography (HPLC), using a Dionex UltiMate 3000 instrument (Thermo Scientific, Waltham, MA, USA), equipped with a photodiode array detector model DAD-3000 (RS), coupled to an Amazon Speed Ion-Trap mass spectrometer analyzer (AmaZon speed, Bruker, Billerica, MA, USA) with an electrospray ionization source (ESI). The system was controlled using Bruker Daltonics HyStar 3.2 Software with an Ethernet data interface. The separations were performed through a C18 reversed-phase column, 150 mm long, 4.6 mm internal diameter, and with a fixed phase of 2.6 μm particle size (AcuarateTM RP-MS column, Thermo Scientific, USA). The mobile phase consisted of solvent A (Water 99.9%/0.1% formic acid) and solvent B (MeOH), with a flow rate of 0.5 mL/min in a gradient mode. The elution gradient was conducted at a constant flow rate of 0.5 mL/min as follows: 0 min, 100% A; 2–15 min, 93% A; 35 min, 60% A; 45–47 min, 30% A; 57 min, initial conditions until 60 min as a re-equilibration step. The sample volume injected was 10 μL. Detection was performed at a wavelength of 330 and 280 nm, for quinine and caffeine (internal standard), respectively. Caffeine was selected as an internal standard due to its structural and chemical similarities to quinine. Importantly, caffeine elutes at a distinct retention time from quinine, effectively preventing peak overlap and ensuring accurate and precise quantification of quinine in the samples. The mass spectrometry conditions were set to operate in positive ionization mode, with the following parameters: ionization capillary voltage was maintained at 4.500 V; the mass range was specified as *m/z* 100–2000; the nebulizer pressure was adjusted to 26.0 psi; the nitrogen drying gas temperature was set at 200 °C; the flow rate of the dry nitrogen gas was 6.0 L/min; rolling averages were configured to 2 counts; and the number of averages was established at 2, with ion charge control (ICC) activated. Method optimization included the evaluation of linearity, precision, and accuracy. Five quinine standards (3, 25, 50, 75, 100 μg/mL) were prepared from a 2000 μg/mL standard solution. Linearity was assessed by constructing calibration curves, obtaining a determination coefficient (R^2^) of 0.9990, demonstrating the high precision and reliability of the method.

### 4.5. Specificity of the Method

**Figure 4:**
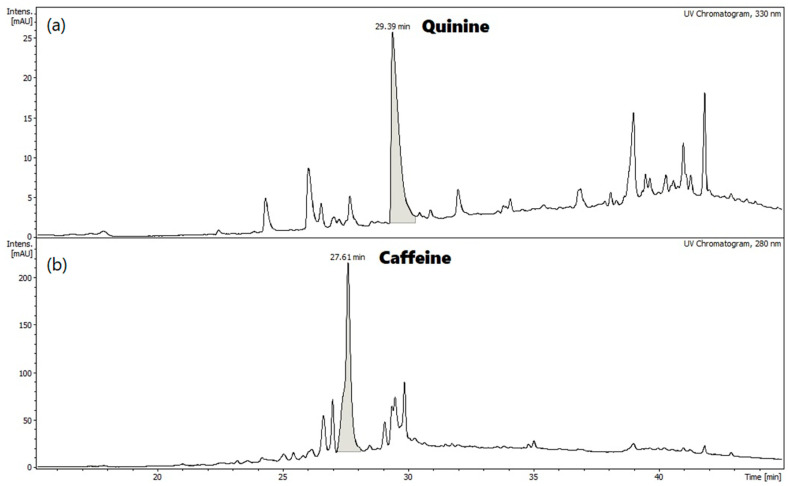
Chromatogram of (**a**) quinine and (**b**) caffeine extracted from Tapichalaca bark using MAE.

The HPLC analytical technique was used to identify the quinine and caffeine (IS) compounds present in the extract obtained under optimal conditions. As can be seen in Figure 4, the retention time (R.T.) for the quinine and caffeine compounds were 29.39 min and 27.61 min, respectively.

**Figure 5:**
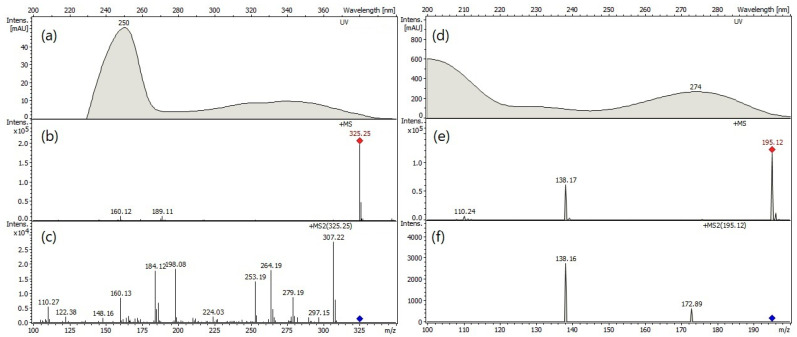
(**a**) UV absorption spectrum of quinine; (**b**) ESI-MS spectrum of quinine; (**c**) ESI-MS^2^ spectrum of quinine; (**d**) UV absorption spectrum of caffeine; (**e**) ESI-MS spectrum of caffeine; (**f**) ESI-MS^2^ spectrum of caffeine.

The molecular ion peak corresponding to quinine has a molecular weight of 325.25 [M+H]^+^ (Figure 5b). While the molecular ion peak of caffeine has a weight of 195.12 [M+H]^+^ (Figure 5e).

### 4.6. Linearity of the Method

**Figure 6:**
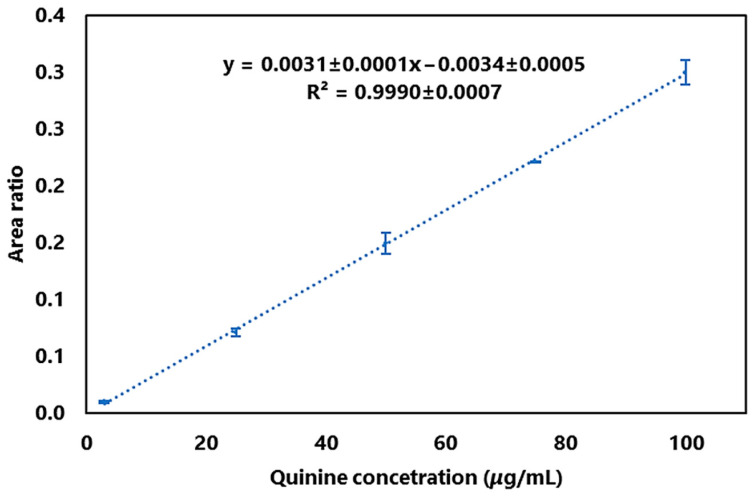
Calibration curve of quinine.

The linearity of the method was developed by means of calibration curves, in relation to the concentration and the area ratio, which are obtained by dividing the area of quinine and caffeine. Using the values of the calibration curves, the equations of the lines were determined (Figure 6), giving an average coefficient of determination of 0.9990 ± 0.0007. The limit of detection for quinine was established at 1 μg/mL, based on a signal-to-noise ratio of 3. Similarly, the limit of quantification was determined to be 3 μg/mL, representing the lowest concentration at which linearity was maintained.

### 4.7. Statistical Analysis

The statistical analysis was performed using Minitab 16 software (State College, PA, USA). Response surface methodology (RSM) was employed to optimize the extraction conditions for both microwave-assisted extraction (MAE) and ultrasound-assisted extraction (UAE), assessing the effect of variables such as extraction time, temperature, and solvent concentration on quinine yield. These analyses facilitated the evaluation of the efficiency of each method and the identification of optimal experimental conditions to achieve the maximum concentration of quinine. Additionally, an analysis of variance (ANOVA) with a 95% confidence interval was conducted to determine whether significant differences existed among the quinine concentrations obtained from different samples. Following this, Tukey’s test was applied to pinpoint specific groups with statistically significant differences.

## 5. Conclusions

This study successfully optimized two advanced extraction methods, microwave-assisted extraction (MAE) and ultrasound-assisted extraction (UAE), for the efficient and rapid recovery of quinine from the *Cinchona officinalis* bark collected from specific locations in Ecuador. Among the evaluated parts of the plant, the bark consistently yielded the highest quinine concentration, highlighting its relevance as a primary source for alkaloid extraction in this species. The optimized conditions for MAE (65% EtOH, 130 °C, 34 min) achieved a maximum yield of 6.30 ± 0.19 mg/g, while UAE (61% EtOH, 25 °C, 15 min) provided a faster but slightly lower yield of 2.81 ± 0.04 mg/g. These findings demonstrate the advantages of MAE and UAE over conventional methods like Soxhlet extraction in terms of time efficiency and sustainability. The use of EtOH–water as an extraction solvent proved effective due to its intermediate polarity, facilitating the selective extraction of quinine while minimizing the co-extraction of unwanted compounds. Additionally, the study underscores the importance of optimizing parameters such as temperature and time to improve extraction efficiency while minimizing compound degradation. The quantification of quinine using high-performance liquid chromatography (HPLC) coupled with advanced detection methods confirmed the reliability and reproducibility of the results. However, it is important to note that these findings are specific to *C. officinalis* collected from the studied locations and may not be directly applicable to other species or regions due to environmental and species-level variability.

While this study provides a valuable framework for optimizing quinine extraction from *C. officinalis* and demonstrates the potential of green chemistry principles in pharmaceutical applications, further research is needed to explore the scalability of these methods and their applicability to other species and contexts. The methods optimized here represent a step toward the sustainable use and valorization of *C. officinalis* byproducts, contributing to the broader development of plant-derived pharmaceuticals.

## Figures and Tables

**Table 1:** Quinine concentrations in the different parts studied and cultivation places in the MAE extraction’s optimal conditions.

Sample	Collection Location	Quinine (mg/g)
Bark	Palanda	<LLD
	Tapichalaca	6.30 ± 0.19 ^a^
	Villonaco	3.93 ± 0.11 ^b^
Trunk	Palanda	<LLD
	Tapichalaca	1.95 ± 0.05 ^c^
	Villonaco	0.12 ± 0.03 ^d^
Leaves	Palanda	<LLD
	Tapichalaca	<LLD
	Villonaco	<LLD

Different lowercase letters indicate significant differences between the means according to ANOVA and Tukey tests (*p* < 0.05). The letter “a” represents the highest value. LLD: limit of detection.

**Table 2:** Design of Box–Behnken response surface for quinine extraction by microwave-assisted extraction (MAE) and ultrasound-assisted extraction (UAE).

Microwave-Assisted Extraction (MAE)				Ultrasound-Assisted Extraction (UAE)			
EtOH (%)	Temperature (ºC)	Time (min)	Quinine (mg/g)	EtOH (%)	Temperature (ºC)	Time (min)	Quinine (mg/g)
100	100	15	1.96	50	25	60	1.58
50	100	37.5	3.62	50	70	60	1.76
100	50	37.5	0.64	100	47.5	60	0.81
0	50	37.5	0.14	50	47.5	37.5	1.66
50	150	15	3.29	0	47.5	60	0.30
100	100	60	1.70	100	70	37.5	1.02
50	50	60	0.79	50	47.5	37.5	1.37
0	100	60	0.41	100	47.5	15	1.00
0	150	37.5	0.35	50	25	15	2.06
0	100	15	0.45	0	25	37.5	0.23
100	150	37.5	3.02	0	47.5	15	0.27
50	100	37.5	3.53	100	25	37.5	1.04
50	100	37.5	3.33	0	70	37.5	0.31
50	50	15	3.12	50	47.5	37.5	1.51
50	150	60	2.76	50	70	15	1.42

**Table 3:** Analysis of variance of response surface design for quinine: (a) microwave-assisted extraction and (b) ultrasound-assisted extraction.

	Parameter	GL	SC Adjustment	MC Adjustment	*f*-Value	*p*-Value
(a) Microwave-assisted extraction (MAE)	Model	9	24.8341	2.7593	15.23	0.004
	Linear	3	8.4923	2.8308	15.63	0.006
	EtOH	1	4.4603	4.4603	24.62	0.004
	Temp	1	2.7871	2.7871	15.39	0.011
	Time	1	1.2449	1.2449	6.87	0.047
	Square	3	14.3250	4.7750	26.36	0.002
	EtOH × EtOH	1	13.4371	13.4371	74.18	0.000
	Temp × Temp	1	1.1048	1.1048	6.10	0.057
	Time × Time	1	0.7658	0.7658	4.23	0.095
	2-Way Interaction	3	2.0167	0.6722	3.71	0.096
	EtOH × Temp	1	1.1827	1.1827	6.53	0.051
	EtOH × Time	1	0.0129	0.0129	0.07	0.800
	Temp × Time	1	0.8211	0.8211	4.53	0.087
	Error	5	0.9057	0.1811		
	Lack-of-Fit	3	0.8620	0.2873	13.14	0.072
	Pure Error	2	0.0437	0.0219		
	Total	14	25.7398			
(b) Ultrasound-assisted extraction (UAE)	Model	9	4.98309	0.55368	32.93	0.001
	Linear	3	0.7231	0.24103	14.34	0.007
	EtOH	1	0.7121	0.7121	42.35	0.001
	Temp	1	0.00428	0.00428	0.25	0.635
	Time	1	0.00456	0.00456	0.27	0.625
	Square	3	3.82003	1.27334	75.73	0.000
	EtOH × EtOH	1	3.6072	3.6072	214.54	0.000
	Temp × Temp	1	0.05571	0.05571	3.31	0.128
	Time × Time	1	0.01865	0.01865	1.11	0.340
	2-Way Interaction	3	0.18283	0.06094	3.62	0.100
	EtOH × Temp	1	0.00272	0.00272	0.16	0.704
	EtOH × Time	1	0.01123	0.01123	0.67	0.451
	Temp × Time	1	0.16888	0.16888	10.04	0.025
	Error	5	0.08407	0.01681		
	Lack-of-Fit	3	0.04275	0.01425	0.69	0.637
	Pure Error	2	0.04132	0.02066		
	Total	14	5.06716			

## Data Availability

The data is available through the article.
